# Associations between serum biomarkers and non-alcoholic liver disease: Results of a clinical study of Mediterranean patients with obesity

**DOI:** 10.3389/fnut.2022.1002669

**Published:** 2022-09-08

**Authors:** Sara De Nucci, Fabio Castellana, Roberta Zupo, Luisa Lampignano, Martina Di Chito, Roberta Rinaldi, Vito Giannuzzi, Raffaele Cozzolongo, Giuseppina Piazzolla, Gianluigi Giannelli, Rodolfo Sardone, Giovanni De Pergola

**Affiliations:** ^1^Unit of Geriatrics and Internal Medicine, National Institute of Gastroenterology–IRCCS “Saverio de Bellis”, Castellana Grotte, Italy; ^2^Unit of Data Sciences and Technology Innovation for Population Health, National Institute of Gastroenterology–IRCCS “Saverio de Bellis”, Castellana Grotte, Italy; ^3^Department of Gastroenterology, National Institute of Gastroenterology-IRCCS “Saverio de Bellis”, Castellana Grotte, Italy; ^4^Interdisciplinary Department of Medicine, Internal Medicine Unit, University of Bari, Bari, Italy; ^5^Scientific Direction, National Institute of Gastroenterology-IRCCS “Saverio de Bellis”, Castellana Grotte, Italy

**Keywords:** fatty liver, NAFLD, transient elastography, obesity, cross sectional analysis

## Abstract

**Background:**

Transient elastography is an ultrasound-based method to detect non-alcoholic fatty liver disease (NAFLD). Despite the simultaneously rising prevalence of fatty liver and metabolic disease, further information about metabolic risk indicators of fatty liver is still necessary.

**Methods:**

A Southern Italian population sample with obesity (*N* = 87) was cross-sectionally explored for associations among the presence of NAFLD, assessed by FibroScan, and clinical, biochemical and anthropometric parameters. Inclusion criteria were age >18 years, BMI ≥ 25 kg/m^2^, no ongoing supplemental or drug therapy, including oral contraceptives or osteoporosis medications; exclusion criteria were pregnancy, endocrinological diseases, cardiovascular diseases, neoplasia, renal or hepatic failure, hereditary thrombocytopenia, hepatitis B (HBV) or hepatitis C virus (HCV) infection, and excess alcohol consumption.

**Results:**

The study sample featured a female predominance (67%, *N* = 60), age range 18–64 years, and 40% prevalence of NAFLD, in accordance with the fibroscan-measured controlled attenuation parameter (CAP) threshold value above 302 dB/m. Males were slightly more frequently affected by NAFLD (51.4% vs. 48.6%, *p* = 0.01). Insulin levels, insulin resistance (quantified by HOMA-IR), diastolic blood pressure, BMI, visceral adipose tissue (VAT), and waist circumference were significantly higher in the NAFLD subset compared to their counterparts (*p* < 0.01, *p* < 0.01, *p* = 0.05, *p* < 0.01, *p* < 0.01, *p* < 0.01, respectively). Uric acid (*p* < 0.01) also showed a positive trend in the NAFLD group. Other liver steatosis parameters, measured by stiffness (*p* < 0.01), fatty liver index (FLI) (*p* < 0.01) and FibroScan-AST (FAST) (*p* < 0.01), were also significantly greater in the NAFLD group. In three nested linear regression models built to assess associations between CAP values and serum uric acid levels, a single unit increase in uricemia indicated a CAP increase by 14 dB/m, after adjusting for confounders (coefficient: 14.07, 95% CI 0.6–27.54).

**Conclusions:**

Clinical-metabolic screening for NAFLD cannot ignore uricemia, especially in patients with obesity.

## Introduction

Liver disease has emerged as one of the most troubling epidemiologic health outcomes of non-communicable disease burdens over the past decade (1). Now-a-days, liver epidemiology is shifting from viral hepatitis, that is well addressed by vaccines or drugs, to fatty liver disease, likely due to the increasing prevalence of metabolic syndrome. In this context, non-alcoholic fatty liver disease (NAFLD) is the most common cause of chronic liver disease in developed countries, exceeding 25% among European adults (2). The highest prevalence has been reported in the Middle East and South America, reaching 70% in populations with obesity and diabetes (3, 4). Individuals with metabolic syndrome or its components are more frequently affected by fatty liver disease, clinically described as a spectrum of liver disease combined with metabolic and cardiovascular disorders (5). NAFLD is closely linked to abdominal obesity, diabetes, and dyslipidemia, as well as with rising levels of clinical and molecular markers of insulin resistance and metabolic syndrome. To the point that, a group of hepatologists from around the world have lately suggested renaming NAFLD as MAFLD (metabolic associated fatty liver disease) (6).

Hepatic steatosis is defined as over 5% liver fat accumulation, not attributable to causes such as excessive alcohol consumption, viral infections, or medications (7, 8). Subcategories of NAFLD include histologic evidence of hepatic steatosis or non-alcoholic steatohepatitis (NASH), a condition of fat accumulation associated with lobular inflammation, with or without fibrosis (9). The prognosis of NAFLD can predict the risk of developing cirrhosis and hepatocellular carcinoma, but the cardiometabolic components are the leading causes of morbidity in these patients (5, 10).

However, since fatty liver is frequently a late-diagnosed and asymptomatic condition in developed countries, morbidity and mortality rates attributable to fatty liver are progressively increasing. In this worsening perspective, obesity phenotypes with excess visceral fat, that account for nearly 80% of fatty liver phenotypes, are more likely to favor liver injury (11). As regards the mechanisms responsible, an increased VAT release of proinflammatory cytokines and adipokines and the delivery of free fatty acids (FFA) into the portal and systemic circulation are the main pathogenic processes underlying NAFLD (12, 13).

For the diagnosis of NAFLD, staging and clinical management, the gold standard, i.e., liver biopsy, is too invasive for widespread use in clinical practice and, therefore, a non-invasive approach is recommended. Serum biomarkers include predictive models such as the hepatic steatosis index (HSI) (14) and the fatty liver index (FLI) (15). A large body of research has endorsed these scores, although they fail to provide further information about individuals with NAFLD. Our recent meta-analysis suggested that the FLI performs well in stratifying the risk of NAFLD but has less efficacy in excluding or diagnosing the condition (16). Instead, transient elastography (FibroScan) is a widely known ultrasound-based technique for use in clinical practice, supporting accurate and reliable assessment of liver steatosis across different populations (17, 18).

The present clinical cross-sectional study targeted a Southern Italian population with overweight and obesity, with the aim of investigating the association between the presence of NAFLD, assessed by the FibroScan ultrasound device, and clinical, biochemical, and anthropometric parameters.

## Materials and methods

### Study population and design

**Figure 1:**
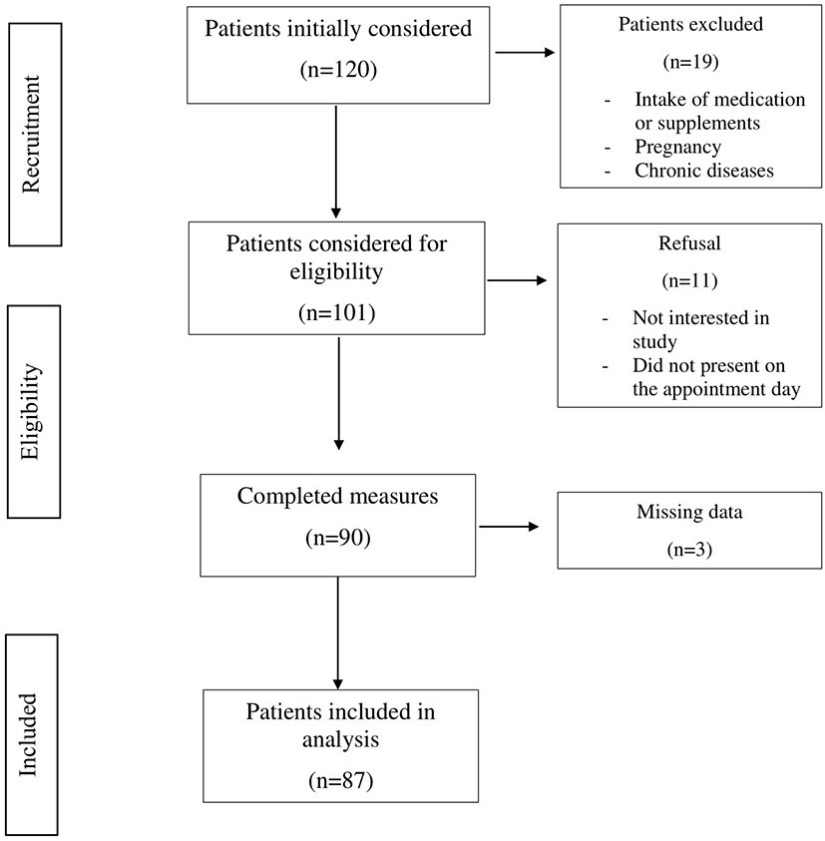
Flowchart of the sample screening process.

Patients were recruited from January to March 2022 at the National Institute of Gastroenterology “Saverio De Bellis” Research Hospital Outpatients Clinic of Internal Medicine and Geriatrics (Castellana Grotte, Bari, Apulia, Italy). Inclusion criteria were age over 18 years, BMI ≥ 25 kg/m^2^, no ongoing integrative or drug therapy, including oral contraceptives or osteoporosis medications. Exclusion criteria were pregnancy, any endocrinological disease (i.e., diabetes mellitus, hypo- or hyperthyroidism, hypopituitarism), chronic inflammatory disease, stable hypertension, angina pectoris, a history of stroke, transient ischemic attack, atrial fibrillation, heart attack, congenital heart disease, any major malignancy, renal or hepatic insufficiency, hereditary thrombocytopenia, hepatitis B (HBV) or hepatitis C virus (HCV) infection, and excessive alcohol consumption. In accordance with American and European guidelines (7, 8), patients who consumed more than two (male) or one (female) glass of alcohol per day were excluded. After excluding all patients not satisfying the inclusion criteria, the final study population consisted of 87 patients (60 females and 27 males). A summary flow diagram of the population screening process is shown in Figure 1. The study protocol complied with the principles of the Declaration of Helsinki and was approved by the Ethics Committee of the National Institute of Gastroenterology “S. De Bellis” Research Hospital (protocol no. 173/2021). All participants gave prior informed consent to enrollment.

### Clinical examination and fluid biomarkers collection

At baseline, all patients underwent a thorough examination of metabolic and biochemical markers. An experienced physician (GDP) conducted an interview probing medical history and lifestyle. Patients who consumed excessive amounts of alcohol, or with viral hepatitis or the human immunodeficiency virus, were excluded. Daily alcohol consumption was assessed through direct questions during the medical history, as follows: “*Do you drink more than two glasses of alcohol per day?”* for males and “*Do you drink more than one glass of alcohol per day?”* for females, following American and European daily alcohol consumption guidelines (7, 8), that suggest a threshold of 20 g/day in females and 30 g/day in males. Extemporaneous outpatients diastolic (DBP) and systolic (SBP) pressure was measured three times, in a sitting position using an OMRON M6 automated Blood Pressure monitor. After overnight fasting, blood samples were collected from 08:00 to 09:00 AM. The blood cell count was measured using a Beckman–Coulter Coulter Hematology analyzer (Brea, CA). Serum levels of fasting plasma glucose (FPG), hemoglobin A1c (HbA1c), insulin, total cholesterol, high-density lipoprotein (HDL) cholesterol, triglycerides, uric acid, and liver markers were measured. Serum insulin concentrations were determined by radioimmunoassay (Behring, Scoppito, Italy), examining duplicate samples. Serum concentrations of TSH, FT3, and FT4 were evaluated using a competitive luminometric method based on the solid-phase antigen luminescent technology (SPALT) principle (LIAISON FT3, FT4, TSH, DiaSorin, Saluggia, Italy). Fasting plasma glucose concentrations were assessed using the glucose oxidase method (Sclavus, Siena, Italy), and plasma lipid concentrations (triglycerides, total cholesterol, and HDL cholesterol) were quantified with an automated colorimetric approach (Hitachi; Boehringer Mannheim, Mannheim, Germany). Glycated hemoglobin (HbA1c) was determined frequently with an Architect c8000 chemical analyzer (Abbott). Serum uric acid was measured by the URICASE/POD method implemented on an autoanalyzer (Boehringer Mannheim). Amino transferase and γ-glutamyl transpeptidase (γGT) were measured with standard routine laboratory methods, and creatinine with an automated system (UniCel Integrated Workstations DxC 660i, Beckman Coulter, Fullerton, CA, USA). Low-density lipoprotein (LDL) cholesterol was calculated using the Friedewald equation (19). Quantitative analysis of serum ferritin was made with DxI/Access using Access Ferritin Reagent Packs (Beckman-Coulter AB, Bromma, Sweden). Serum insulin concentrations were measured by radioimmunoassay (Behring, Scop-pito, Italy) and serum 25 (OH) vitamin D by chemiluminescence (Diasorin Inc, Stillwater, OK, USA). Insulin resistance was assessed using the Homeostasis Model Assessment–Insulin Resistance (HOMA-IR) method (20).

### Anthropometric assessment

Two qualified nutritionists (SDN, RZ), trained to conduct clinical procedures consistently, collected all anthropometric measurements, with patients wearing light clothes and barefoot. Variables were all collected simultaneously between 7.00–10.00 AM after overnight fasting. Height was measured to the nearest 0.5 cm using a wall-mounted stadiometer (Seca 711; Seca, Hamburg, Germany). Body weight was determined to the nearest 0.1 kg using a calibrated balance beam scale (Seca 711; Seca, Hamburg, Germany). BMI was calculated by dividing body weight (Kg) by the square of height (m^2^) and classified according to World Health Organization criteria as normal weight (18.5–24.9 kg/m^2^), overweight (25.0–29.9 kg/m^2^), grade I obesity (30.0–34.9 kg/m^2^), grade II obesity (35.0–39.9 kg/m^2^), and grade III obesity (≥40.0 kg/m^2^) (18). Waist circumference (WC) was measured at the narrowest part of the abdomen or in the area between the tenth rib and the iliac crest (minimum circumference).

Bioelectrical Impedance Analysis (BIA) was performed using a single-frequency bioimpedance analyzer (BIA-101 analyzer, 50-kHz frequency; Akern Bioresearch, Florence, Italy). The instrument is routinely checked with resistors and capacitors of known values. In accordance with European Society of Parenteral and Enteral Nutrition (ESPEN) guidelines (21), all participants were examined supine with their legs spread slightly apart, after refraining from eating, drinking, exercising for 6 h and drinking alcohol for 24 h before the examination. Shoes and socks were removed, and contact areas were scrubbed with alcohol before electrodes placement. Electrodes (BIATRODES Akern, Florence, Italy) were placed proximal to the phalangeal metacarpal joint on the dorsal surface of the right hand and distal to the transverse arch on the superior surface of the right foot. Sensor electrodes were placed at the midpoint between the distal prominence of the radius and ulna of the right wrist and between the medial and lateral malleoli of the right ankle (22). All measurements were made by a senior nutritionist (SP) under strictly standardized conditions. Whole-body impedance vector components, resistance (R, W) and reactance (Xc, W), were derived and recorded when stable. Then, according to the age, gender, weight and height of each patient, body composition parameters were obtained using the software provided by the manufacturer, including validated (21) predictive equations for total body water (TBW, L), extracellular water (ECW, L), intracellular water (ICW, L), fat-free mass (FFM, kg), MM (kg), Appendicular skeletal muscle mass (ASMM, kg), and body cell mass (BCM, kg). Phase angle (PhA, °) values were also drawn from the reactance ratio vs. electric resistance. A more detailed description of all principles applied to derive bioimpedance measurements has been previously reported (23, 24).

### NAFLD assessment by fibroscan

The controlled attenuation parameter (CAP) algorithm implemented in the FibroScan system was chosen to quantify liver fat by measuring the liver attenuation (dB/m) of an ultrasound beam. The method is straightforward, does not require an in-depth knowledge of B-mode ultrasonography, and is increasingly employed as a point-of-care approach in diagnostic examinations of patients with suspected hepatic steatosis. CAP estimates the degree of ultrasound attenuation due to hepatic fat at the standardized frequency of 3.5 MHz by vibration-controlled transient elastography (VCTE), implemented as FibroScan transient elastography (Echosens, Paris, France) (25). Data trials in patients with obesity suggest that CAP measured with FibroScan is comparable to liver biopsy for the detection and quantification of steatosis (26). NAFLD was diagnosed if CAP exceeded 302 dB/m, previously identified as the optimal cutoff for an accurate diagnosis of ≥5% hepatic steatosis using the Youden index, with a sensitivity of 0.80 (95% confidence interval [CI], 0.75–0.84) and specificity of 0.83 (95% CI, 0.69–0.92) (18). Fibrosis ratings were based on liver stiffness using the VCTE approach. The presence of liver fibrosis was recorded in cases with liver stiffness values exceeding 8.2 kPA, and the absence of NAFLD, in cases with a concomitant CAP of <302 dBm and stiffness of <8.2 kPA.

### Liver disease risk estimation algorithms

Prediction algorithms drawn from the literature were included in the descriptive sample analysis to estimate the risk of NAFLD. The FLI, a modeling approach that incorporates BMI, WC, triglycerides, and γGT, was used to determine the likelihood of developing NAFLD (15). The following equation was applied to perform the calculation: [e 0.953^*^loge (TG) + 0.139^*^BMI + 0.718^*^loge (GGT) + 0.053^*^WC−15.745]/[1 + e 0.953^*^loge (TG) + 0.139^*^BMI + 0.718^*^loge (GGT) + 0.053^*^WC−15.745] ^*^ 100. The FAST Score, a modeling algorithm that includes the measurement of liver stiffness (LSM), CAP, and AST, was used to assess the risk of NASH. The following equation was applied: (e −1.65 + 1.07^*^ln(LSM) + 2.66^*^10^−8*^CAP^3^ – 63.3^*^AST^−1^)/1 + (e −1.65 + 1.07^*^ln(LSM) + 2.66^*^10^−8*^CAP^3^ – 63.3^*^AST^−1^) (27). Lastly, the FIB-4 equation to estimate the risk of liver fibrosis included age, AST, ALT, and platelets, as follows: age [(years) x AST (U/L)]/[(PLT [10(9)/L]) x (ALT [U/L])(1/2)] (28).

### Statistical analysis

**Figure 2:**
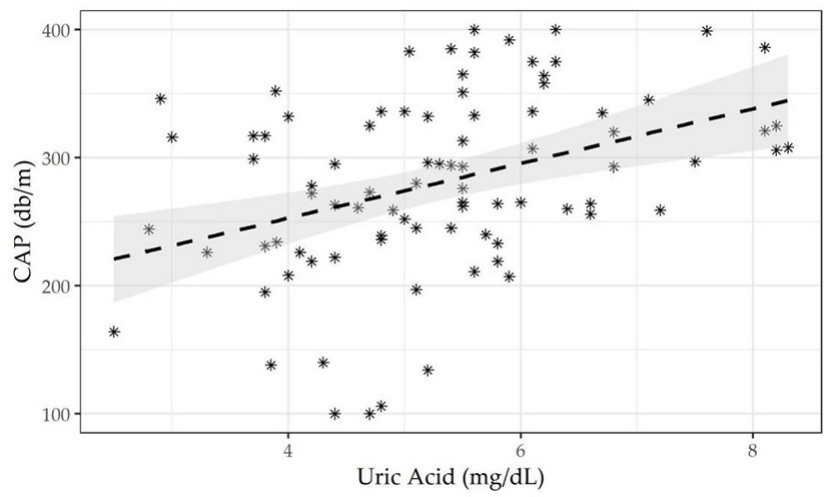
Linear correlation between Controlled Attenuation Parameter (CAP) and serum uric acid.

The entire sample was subdivided by the CAP cutoff according to NAFLD status (presence/absence), to assess differences in frequency and associations with biochemical, sociodemographic, anthropometric, and dietary variables. The Kolmogorov-Smirnov method was used to test the normality of quantitative variable distributions. For continuous measurements, data are expressed as mean ± standard deviations (M±SD), median (min to max), and frequency and percentages (%) for all categorical variables. A statistical approach based on the null hypothesis significance test (NHST) was excluded, focusing on practical variations in effect size across groups owing to the small sample size. The effect size (ES) was used to estimate the prevalence of NAFLD according to the CAP cut-off and other categorical variables and 95% CIs. Differences between continuous variables were computed using Wilxocon's effect size difference between medians. Prevalence differences were used to assess the magnitude of differences between proportions. Three nested linear regression models were run to assess associations between CAP values and serum uric acid levels. The methodological setting of the three models was as follows: first, unadjusted to study the relationship between CAP and uricemia; second, using uric acid, age, gender, and BMI as confounding covariates; third, using the second model plus creatinine, triglycerides, and HOMA index as confounding covariates. Figure 2 shows the linear correlation between CAP and uric acid. Statistical analyses were designed and managed by a senior epidemiologist (RS) and a biostatistician (FC) using RStudio 2021.09.1.3.

## Results

The population examined (*N* = 87) was prevalently female (67%, *N* = 60), and the age range was 18 to 64 years. NAFLD was diagnosed in 35 patients (40% of the whole sample), according to the threshold CAP value above 302, while only ten patients (11.3% of the entire population) showed liver stiffness values exceeding 8.2 kPa, possibly demonstrating fibrosis.

Table 1 summarizes the main differences in clinical-metabolic, anthropometric, and sociodemographic variables according to NAFLD (presence/absence). Males were slightly more frequently affected by NAFLD than females (51.4% vs. 48.6%, *p* = 0.01). The NAFLD patients group showed higher DBP, BMI, and WC (*p* = 0.05, *p* < 0.01, *p* < 0.01, respectively). Among metabolic variables, those linked with insulin effect such as serum HbA1c, insulin levels, and HOMA-IR, were higher in the NAFLD group (*p* = 0.04, *p* < 0.01, *p* < 0.01, respectively). Serum transaminases, including AST (*p* < 0.01) and ALT (*p* = 0.04), and γGT (*p* < 0.01), showed the same trend. Concerning the serum lipid profile, the NAFLD group showed lower HDL (*p* < 0.01) and higher triglyceride levels (*p* = 0.01) as compared to their counterparts. Creatinine (*p* = 0.02), uric acid (*p* < 0.01), and ferritin (*p* < 0.01) followed the same positive trend in the NAFLD group. Liver stiffness (*p* < 0.01), FLI (*p* < 0.01), FAST score (*p* < 0.01), and CAP (*p* < 0.01) were higher in the NAFLD group. Findings from the BIA examination revealed significantly higher levels of FM (*p* = 0.01), FFM (*p* < 0.01), TBW (*p* < 0.01), ECW (*p* < 0.01), and VAT (*p* < 0.01) in the NAFLD group.

**Table 1:** Description of the whole sample according to the Controlled Attenuation Parameter (CAP) level (CAP <302 dB/m/CAP ≥ 302 dB/m).

	**Median**	**Median**	***P* value**
	**(min to max)**	**(min to max)**	
Proportions (%)			
Age (years)	43 (18 to 64)	44 (19 to 62)	0.78
Gender			
Female	43 (82.70)	17 (48.60)	0.01
Male	9 (17.30)	18 (51.40)	
Diastolic blood pressure (mmHg)	77 (55 to 98)	84 (50 to 110)	0.05
Systolic blood pressure (mmHg)	129 (105 to 158)	130 (110 to 165)	0.09
BMI (Body Mass Index) (Kg/m^2^)	31 (25.1 to 47.5)	35.49 (25.8 to 47.33)	**<**0.01
WC (Waist circumference) (cm)	101 (81 to 144)	115 (97 to 141)	**<**0.01
Fasting blood glucose (mg/dl)	93 (76 to 118)	97 (79 to 156)	0.31
HbA1c (%)	5.2 (4.3 to 6.2)	5.45 (4.5 to 7.3)	0.04
HOMA-IR	2.17 (0.45 to 9.27)	3.35 (0.96 to 16.54)	**<**0.01
Insulin (μU/ml)	8.79 (2.3 to 38.3)	14.9 (4.4 to 54.9)	**<**0.01
GGT (U/L)	15 (7 to 93)	23 (9 to 89)	**<**0.01
AST (GOT) (U/L)	21 (10 to 86)	33 (13 to 143)	**<**0.01
ALT (GPT) (U/L)	20 (11 to 47)	22 (13 to 58)	0.04
HDL cholesterol (mg/dl)	52.5 (32 to 109)	45 (30 to 76)	**<**0.01
LDL cholesterol (mg/dl)	140.5 (76 to 219)	134 (71 to 224)	0.62
Total cholesterol (mg/dl)	215.5 (152 to 295)	198 (140 to 319)	0.23
Triglycerides (mg/dl)	85 (25 to 266)	117 (48 to 271)	0.01
Creatinine (mg/dl)	0.7 (0.5 to 1.11)	0.8 (0.4 to 1.23)	0.02
Uric Acid (mg/dl)	4.95 (2.5 to 7.5)	5.6 (2.9 to 8.3)	**<**0.01
Ferritin (ng/ml)	51.7 (3 to 226)	103 (10 to 603)	**<**0.01
25-Hydroxyvitamin D (ng/ml)	19.1 (8.3 to 49.4)	19.4 (4 to 36.6)	0.89
WBC (White Blood Cells) (10^3^cells/mm^3^)	6.53 (4.01 to 11)	6.84 (4.24 to 10.4)	0.33
FAST score (Fibroscan-AST score)	0.05 (0 to 0.45)	0.19 (0.02 to 0.78)	**<**0.01
CAP (Controlled Attenuation Parameter) (dB/m)	245 (100 to 299)	336 (306 to 400)	**<**0.01
Liver stiffness (kPa)	4.7 (2.3 to 10.4)	5.6 (2.7 to 28.5)	**<**0.01
TSH (μU/ml)	1.62 (0.43 to 4.97)	2.02 (0.28 to 6.45)	0.13
FT3 (pg/ml)	3.28 (2.6 to 4.24)	3.48 (2.71 to 4.79)	0.06
FT4 (pg/ml)	9.2 (6.5 to 15.4)	9.5 (6.8 to 14.3)	0.16
FIB-4 (Fibrosis-4 scoring system)	0.64 (0.24 to 1.68)	0.65 (0.23 to 1.5)	0.87
FLI (Fatty Liver Index)	57 (14 to 99)	87 (42 to 100)	**<**0.01
FLI >60%	24 (46.20)	30 (85.70)	
FFM (Free Fat Mass) (Kg)	51.32 (39.2 to 77.8)	63.37 (37.2 to 99.62)	**<**0.01
FFMI (Free Fat Mass Index) (Kg/m^2^)	19.15 (14.6 to 55)	21.2 (11.3 to 28.5)	**<**0.01
FM (Fat Mass) (Kg)	32.21 (21.06 to 63.78)	39.5 (22.4 to 61.56)	0.01
FMI (Fat Mass Index) (Kg/m^2^)	11.9 (6.6 to 45)	12.7 (6.3 to 25.4)	0.51
SMM (Skeletal Muscle Mass) (Kg)	24.95 (18.6 to 39.9)	31.8 (17.8 to 51.8)	**<**0.01
TBW (Total Body Water) (L)	38.45 (29.6 to 57.5)	47.7 (27.9 to 74.2)	**<**0.01
ECW (Extra Cellular Water) (L)	17.05 (13.4 to 24.7)	20.6 (12.8 to 31.1)	**<**0.01
VAT (Visceral Adipose Tissue) (L)	2.3 (0.1 to 7.91)	4.46 (1.6 to 11)	**<**0.01

*N*: 87.

All data are shown as median (min to max) and as *n* (%) for proportions.

Wilcoxon U test for independent samples for continuous variables and Chi squared test for categorical ones.

Bold and underlined values indicate the significance of the data.

Supplementary Table S1 presents the Spearman correlation matrix findings between CAP values and all other parameters examined in this study. CAP levels were directly associated with BMI, WC, systolic blood pressure, HOMA index, insulin levels, triglycerides, uric acid, AST, ALT, γGT, creatinine, and ferritin, and negatively correlated with HDL concentrations. These findings were used to select confounding variables in order to build more accurate regression models.

Table 2 shows three nested linear regression model findings run to assess associations between CAP values and serum uric acid levels. A single unit increase in uricemia showed an increase in CAP by 14 dB/m, after adjusting for the confounders age, gender, BMI, creatinine, triglycerides, ALT, AST, γGT and HOMA-IR. The scatter plot shown in Figure 2 highlights the effect of the positive relationship between uricemia levels and CAP in our sample with obesity (correlation CAP/uricemia 0.33, *p* <0.01).

**Table 2:** Linear regression model on Controlled Attenuation Parameter (CAP) as dependent variable and regressor.

	**Coefficient**	**Stand. Err**.	**CI 95%**	***p***	**Coefficient**	**Stand. Err**.	**CI 95%**	***p***	**Coefficient**	**Stand. Err**.	**CI 95%**	***p***
Uric acid (mg/dl)	21.36	5.5	108.34 to 226.38	**<**0.01	14.79	6.89	1.28 to 28.30	0.03	15.18	6.71	2.02 to 28.34	0.02
Age (years)					0.45	0.55	−0.63 to 1.54	0.41	1.01	0.58	−0.12 to 2.15	0.08
Gender (male)					21.57	18	−15.28 to 58.42	0.25	−4.91	23.05	−50.08 to 40.27	0.83
BMI (Kg/m^2^)					3.21	1.29	0.69 to 5.75	0.01	2.44	1.45	−0.39 to 5.27	0.09
Creatinine (mg/dl)									−17.67	57.33	−130.04 to 94.69	0.76
Triglycerides (mg/dl)									0.09	0.13	−0.16 to 0.35	0.48
HOMA-IR									2.4	3.93	−5.3 to 10.09	0.54
AST (U/L)									−2.41	1.92	−6.18 to 1.36	0.21
ALT (U/L)									1.65	0.77	0.15 to 3.15	0.03

Bold and underlined values indicate the significance of the data.

## Discussion

The present study examined a population of diabetes-free subjects with obesity, performing transient elastography to identify the metabolic markers most strongly associated to NAFLD, identified by a CAP exceeding 302 dB/m, previously indicated as the optimal cutoff for an accurate fatty liver diagnosis by fibroscan (18). Key findings were the male predominance in the NAFLD subset, as well as higher BMI, abdominal fat, and insulin resistance (quantified by HOMA-IR), and increased blood insulin, HbA1c, triglycerides, uric acid, transaminases, γGT, ferritin, and creatinine levels and lower serum HDL cholesterol concentrations. NAFLD notably afflicts males more than females in accordance with the scientific evidence. In fact, the protective effect of estrogens likely influences this result, as well as the greater male inclination to abdominal obesity, directly related to NAFLD (29).

The metabolic profile of the NAFLD subset was strongly aligned to the body composition, characterized by a marked excess of fat mass and VAT. In this context, a report from the Dionysos and Nutrition Liver Study observed a 6-fold increased risk of NAFLD in subjects with abdominal obesity, regardless of altered liver enzymes (30). The latest guidelines from the Italian Association for the Study of the Liver (AISF), the Italian Diabetes Society (SID), and the Italian Obesity Society (SIO) report NAFLD in 54–90% of individuals with obesity (31). Expert authors have advised physicians to recommend weight loss through intensive, structured lifestyle programs conducted under specialist supervision and/or the use of pharmacotherapy and/or bariatric surgery in NAFLD patients with obesity, to reduce the severity of liver disease (31).

The association between derangement of glucose metabolism and NAFLD is widely discussed in the literature, and is summarily explained by a conceivable two-way relationship. On one hand, systemic insulin resistance may promote increased free fatty acid flux from peripheral tissues to the liver, leading to the development and progression of NAFLD, even before the onset of diabetes (32). Additionally, the liver has a de novo increase in lipogenesis, which contributes to triglyceride accumulation. Hepatic steatosis and steatohepatitis are promoted by the lipotoxicity of ceramides and diacylglycerol, a condition that is well documented in T2DM. Reactive oxygen species generated by mitochondrial malfunction cause beta-cell destruction and hepatic inflammation, accelerating the development of both NAFLD and T2DM (33). On the other hand, the NAFLD pathophysiology also leads to changes in the hepatic secretion of proteins, lipids, other metabolites, and miRNAs, which may disrupt the liver, muscle, adipose tissue, and pancreatic metabolism, thus promoting insulin resistance (34). Moreover, it should be noted that studies on the link between genetically determined liver steatosis and insulin resistance continue to yield conflicting results (35, 36).

The rise in creatinine can be based on the assumption that patients with NAFLD have a higher prevalence of CKD regardless of their age, gender, body mass index (BMI), or other confounding variables. Possible mechanisms include the systemic release of pathogenic mediators from the steatosic and inflamed liver, such as increased reactive oxygen species, advanced glycation end products, C-reactive protein (CRP), pro-inflammatory, profibrogenic, and inflammatory mediators. Because NAFLD and CKD share risk factors, both liver and kidney injury may be caused by obesity-related disease mechanisms, such as lipotoxicity, oxidative stress, increased pro-inflammatory cytokine production, and RAAS axis activation (37).

Hyperferritinemia in NAFLD patients is driven by hepatic inflammation and adiponectin, which is a marker of insulin resistance. Serum iron changes are common in adult NAFLD, which is characterized by increased ferritin levels and normal transferrin saturation. Furthermore, in NAFLD, serum ferritin has been linked to the iron-regulating hormone, hepcidin, and hepatic iron levels (38).

As regards uric acid, a single unit increase in uricemia was shown to raise the CAP by 14 dB/m, and this association was independent of confounding factors. As far as the underlying mechanisms are concerned, high levels of serum uric acid may facilitate the onset of NAFLD through multifaceted pathways. Firstly, uric acid induces fat accumulation *via* the generation of endoplasmic reticulum stress and SREBP-1c activation in hepatocytes (39). Secondly, hyperuricemic subjects are more prone to develop fructose-induced fatty liver, since uric acid up-regulates fructokoinase (KHK) expression in human hepatocytes, thus amplifying the lipogenic effect of fructose (40). Thirdly, higher levels of uric acid may foster the development of insulin resistance by reducing endothelial nitric oxide bioavailability and cell supply (41) and, as previously reported (32), insulin resistance *per se* may be responsible for liver steatosis.

### Strength and limitations

This research features the following strengths. The methodological plausibility and originality of this study findings may be credited to the statistical analysis performed on a consistent cohort from southern Italy, sharing similar traits, and including only individuals taking neither medication nor supplements, thus avoiding any possible pharmacological interference. NAFLD was estimated using a FibroScan controlled attenuation parameter algorithm, still the only guidelines-recommended tool to assess hepatic steatosis when high-cost imaging and biopsy are not available, and widely acknowledged as a point-of-care approach in diagnostic examinations of patients with suspected hepatic steatosis. Some limitations must also be acknowledged. Due to the cross-sectional design, we cannot appreciate any temporal nature of the associations, so prospective observations are required to elucidate any causal relationship. A thorough lifestyle analysis, including genetics and eating habits, would also have been useful, added to the database and to the findings. Moreover, since this is a monocentric study, the results cannot be completely generalized.

## Conclusions

We used transient elastography to investigate the association among the presence of NAFLD and clinical, biochemical, and anthropometric parameters in a sample of subjects with overweight and obesity. The metabolic markers found to best illustrate NAFLD are: high BMI and abdominal fat, insulin resistance, and increased blood insulin, HbA1c, triglycerides, transaminases, GT, ferritin, creatinine, and uric acid levels, in contrast to a reduction in serum HDL cholesterol. It was a particularly original finding worthy of note that a single unit increase in uricemia was associated to a rise of CAP by 14 dB/m, paralleling an increase of liver steatosis. On this basis, more attention should be paid to uricemia as a possible marker of an increased risk of developing fatty liver disease in subjects with obesity.

## Data availability statement

The original contributions presented in the study are included in the article/Supplementary material, further inquiries can be directed to the corresponding author/s.

## Ethics statement

The studies involving human participants were reviewed and approved by Institutional Review Board (or Ethics Committee) of National Institute of Gastroenterology “Saverio de Bellis”, Research Hospital. The patients/participants provided their written informed consent to participate in this study.

## Author contributions

SD, RZ, FC, GD, RS, GG, and GP: conceptualization, research, resource provision, data collection, writing original version, and visualization. MD, RR, LL, RC, VG, and SD: research and data collection. GG: funding. RS and RZ: conceptualization, validation, review, and correction. All authors: conceptualization, validation, and visualization. All authors contributed to the article and approved the submitted version.

## Funding

Italian Ministry of Health with Ricerca Corrente 2020 funds.

## Conflict of interest

The authors declare that the research was conducted in the absence of any commercial or financial relationships that could be construed as a potential conflict of interest.

## Publisher's note

All claims expressed in this article are solely those of the authors and do not necessarily represent those of their affiliated organizations, or those of the publisher, the editors and the reviewers. Any product that may be evaluated in this article, or claim that may be made by its manufacturer, is not guaranteed or endorsed by the publisher.
