# Corrigendum: Associations between serum biomarkers and non-alcoholic liver disease: results of a clinical study of Mediterranean patients with obesity

**DOI:** 10.3389/fnut.2023.1198144

**Published:** 2023-05-04

**Authors:** Sara De Nucci, Fabio Castellana, Roberta Zupo, Luisa Lampignano, Martina Di Chito, Roberta Rinaldi, Vito Giannuzzi, Raffaele Cozzolongo, Giuseppina Piazzolla, Gianluigi Giannelli, Rodolfo Sardone, Giovanni De Pergola

**Affiliations:** ^1^Unit of Geriatrics and Internal Medicine, National Institute of Gastroenterology-IRCCS “Saverio de Bellis”, Castellana Grotte, Italy; ^2^Unit of Data Sciences and Technology Innovation for Population Health, National Institute of Gastroenterology-IRCCS “Saverio de Bellis”, Castellana Grotte, Italy; ^3^Department of Gastroenterology, National Institute of Gastroenterology-IRCCS “Saverio de Bellis”, Castellana Grotte, Italy; ^4^Interdisciplinary Department of Medicine, Internal Medicine Unit, University of Bari, Bari, Italy; ^5^Scientific Direction, National Institute of Gastroenterology-IRCCS “Saverio de Bellis”, Castellana Grotte, Italy

**Keywords:** fatty liver, NAFLD, transient elastography, obesity, cross sectional analysis

In the published article, there was an error in the affiliation list.

Instead of “Unit of Geriatrics and Internal Medicine, National Institute of Gastroenterology “Saverio de Bellis”, Research Hospital, Bari, Italy”, authors Sara De Nucci, Martina Di Chito, Roberta Rinaldi and Giovanni De Pergola should be affiliated to “Unit of Geriatrics and Internal Medicine, National Institute of Gastroenterology-IRCCS “Saverio de Bellis”, Castellana Grotte, Italy”.

Instead of “Unit of Data Sciences and Technology Innovation for Population Health, National Institute of Gastroenterology “Saverio de Bellis”, Research Hospital, Bari, Italy”, authors Fabio Castellana, Roberta Zupo, Luisa Lampignano and Rodolfo Sardone should be affiliated to “Unit of Data Sciences and Technology Innovation for Population Health, National Institute of Gastroenterology-IRCCS “Saverio de Bellis”, Castellana Grotte, Italy”.

Instead of “Department of Gastroenterology, National Institute of Gastroenterology “Saverio de Bellis”, Research Hospital, Bari, Italy”, authors Vito Giannuzzi and Raffaele Cozzolongo should be affiliated to “Department of Gastroenterology, National Institute of Gastroenterology-IRCCS “Saverio de Bellis”, Castellana Grotte, Italy”.

Instead of “Scientific Direction, National Institute of Gastroenterology “Saverio de Bellis”, Research Hospital, Bari, Italy”, author Gianluigi Giannelli should be affiliated to “Scientific Direction, National Institute of Gastroenterology-IRCCS “Saverio de Bellis”, Castellana Grotte, Italy”.

The authors apologize for this error and state that this does not change the scientific conclusions of the article in any way. The original article has been updated.

