# Supplementary material for: Associations between serum biomarkers and non-alcoholic liver disease: Results of a clinical study of Mediterranean patients with obesity
Source: Front Nutr. 2022 Sep 8;9:1002669. doi: 10.3389/fnut.2022.1002669 (PMC9493452; doi:10.3389/fnut.2022.1002669)
Supplement: Supplementary file 1 [file Table_1.docx]

**Supplementary Table 1.** Spearman’s correlation matrix.

|  | CAP | |
| --- | --- | --- |
|  | Rho | p |
| Age (Years) | -0.01 | 0.92 |
| FLI | 0.5 | **<0.01** |
| FIB-4 | 0.04 | 0.75 |
| Liver Stiffness | 0.3 | **<0.01** |
| Body Mass Index (BMI) (Kg/m^2^) | 0.36 | **<0.01** |
| Waist Circumference (WC) (cm) | 0.47 | **<0.01** |
| Total Cholesterol (mg/dl) | -0.11 | 0.32 |
| LDL Cholesterol (mg/dl) | -0.04 | 0.73 |
| TSH (µU/ml) | 0.12 | 0.26 |
| FT3 (pg/ml) | 0.16 | 0.14 |
| FT4 (pg/ml) | 0.15 | 0.15 |
| 25-Hydroxyvitamin D (ng/ml) | -0.13 | 0.25 |
| Uric Acid (mg/ml) | 0.4 | **<0.01** |
| Creatine (mg/dl) | 0.24 | **0.03** |
| White blood cells (10^3^cells/mm^3^) | 0.17 | 0.11 |
| Aspartate transaminase (U/L) | 0.24 | **0.02** |
| Alanine amino transferase (U/L) | 0.35 | **<0.01** |
| gamma-Glutamyl transferase (U/L) | 0.4 | **<0.01** |
| Ferritin (ng/ml) | 0.3 | **<0.01** |
| Visceral Adipose Tissue (L) | 0.43 | **<0.01** |
| Fat Mass (Kg) | 0.23 | **0.03** |
| Free-Fat Mass (Kg) | 0.49 | **<0.01** |
| Total Body Water (L) | 0.5 | **<0.01** |
| Fat Mass Index (Kg/m^2^) | 0.09 | 0.39 |
| Free-Fat Mass Index (Kg/m^2^) | 0.41 | **<0.01** |
| Extra-Cellular Water (L) | 0.52 | **<0.01** |
| Skeletal Muscle Mass (Kg) | 0.49 | **<0.01** |
